# A New Role for a Synaptotagmin Protein in Calcium-Dependent Exocytosis

**DOI:** 10.1371/journal.pbio.0020253

**Published:** 2004-06-29

The hardest working molecules in cell biology, proteins abound in a dazzling variety of shapes and sizes to carry out an equally impressive array of tasks. Many proteins function within the cell, while others get shipped out to locations both near and far. Specialized organelles within the cell package the traveling proteins into cargo containers called vesicles. Vesicles move through a highly regulated transportation system until they reach their ultimate destination, either inside or outside the cell, and release their cargo. When vesicles fuse to the plasma membrane and release their cargo outside the cell the process is called exocytosis. Exocytosis allows macromolecules to leave the cell without compromising the structural integrity of its membrane.

“Professional” secretory cells specialize in producing copious quantities of their protein product and sending vesicles packed with their customized issue to the plasma membrane. Once there, the vesicles wait for a signal to fuse with the membrane. The signal most often comes in the form of a transient and localized increase in calcium ion levels.

Over a decade ago, researchers discovered that calcium-triggered exocytosis also occurs in “nonprofessional” secretory cells. In these cells, the process was thought to be important for healing ruptured plasma membrane, though the identity of the vesicles responsible remained unknown. It has been suggested that these vesicles are lysosomes, enzyme-filled organelles that break down waste and extracellular debris ingested by the cells. Sanford Simon, with his colleagues Jyoti Jaiswal and Norma Andrews, previously confirmed that calcium does specifically trigger exocytosis of lysosomes in the “nonprofessional” secretory cells. Calcium-triggered exocytosis is thought to require the services of a family of proteins called synaptotagmins. But the fifteen members of the synaptotagmin family diverge from this job description in various ways, calling its role into question.

Synaptotagmin VII (Syt VII)—the synaptotagmin member expressed on the lysosomes—is present in most tissues in organisms ranging from worms to humans. This protein functions in processes requiring lysosomal exocytosis and during invasion by trypanosome parasites, such as the one that causes Chagas disease. In this issue of *PLoS Biology*, Jaiswal, Simon, and colleagues investigate the molecular mechanisms underlying calcium-triggered lysosomal exocytosis, focusing on the role of Syt VII.

The researchers took cells from two lines of mice: one lacked the functional Syt VII protein and the other produced normal levels of Syt VII. First they labeled the surface and interior cavity of lysosomes in these cells with fluorescent tags and triggered an increase in cells' calcium level; then they watched the behavior of single lysosomes releasing their contents in real time.

Most of the lysosomes from normal cells released only a portion of their contents and had very small fusion pores that remained open to the outside of the cell for only a short time. Interestingly, while proteins in secretory vesicle membranes typically diffuse into the plasma membrane during exocytosis, proteins in the lysosomal membrane stayed near the site of fusion.

In the cells from Syt VII–deficient mice, Simon and colleagues discovered, to their surprise, that this protein isn't necessary for calcium-triggering of lysosomal exocytosis. Calcium-triggered exocytosis not only occurred in these cells, it happened more rapidly than in normal cells. Plus most ofthese deficient lysosomes fused completely and their membrane proteins fully diffused into the plasma membrane. Simon and colleagues argue that these results show that Syt VII restricts rather than facilitates lysosomal exocytosis. It does so by limiting the formation and size of fusion pores and by preventing lysosomal membrane proteins from integrating into the plasma membrane.

